# Robot Programming from Fish Demonstrations

**DOI:** 10.3390/biomimetics8020248

**Published:** 2023-06-10

**Authors:** Claudio Massimo Coppola, James Bradley Strong, Lissa O’Reilly, Sarah Dalesman, Otar Akanyeti

**Affiliations:** 1 Department of Computer Science, Aberystwyth University, Ceredigion SY23 3DB, UK; 2 Department of Life Sciences, Aberystwyth University, Ceredigion SY23 3DA, UKsad31@aber.ac.uk (S.D.)

**Keywords:** bio-inspired robotics, learning from demonstration, robot programming, fuzzy controller, ANN controller

## Abstract

Fish are capable of learning complex relations found in their surroundings, and harnessing their knowledge may help to improve the autonomy and adaptability of robots. Here, we propose a novel learning from demonstration framework to generate fish-inspired robot control programs with as little human intervention as possible. The framework consists of six core modules: (1) task demonstration, (2) fish tracking, (3) analysis of fish trajectories, (4) acquisition of robot training data, (5) generating a perception–action controller, and (6) performance evaluation. We first describe these modules and highlight the key challenges pertaining to each one. We then present an artificial neural network for automatic fish tracking. The network detected fish successfully in 85% of the frames, and in these frames, its average pose estimation error was less than 0.04 body lengths. We finally demonstrate how the framework works through a case study focusing on a cue-based navigation task. Two low-level perception–action controllers were generated through the framework. Their performance was measured using two-dimensional particle simulations and compared against two benchmark controllers, which were programmed manually by a researcher. The fish-inspired controllers had excellent performance when the robot was started from the initial conditions used in fish demonstrations (>96% success rate), outperforming the benchmark controllers by at least 3%. One of them also had an excellent generalisation performance when the robot was started from random initial conditions covering a wider range of starting positions and heading angles (>98% success rate), again outperforming the benchmark controllers by 12%. The positive results highlight the utility of the framework as a research tool to form biological hypotheses on how fish navigate in complex environments and design better robot controllers on the basis of biological findings.

## 1. Introduction

Manual robot programming is an iterative process. Programmers apply their knowledge, experience, and intuition to design and refine the code through a trial-and-error process until the performance of the robot meets the desired requirements. This is a time-consuming, laborious, and difficult task which requires good understanding of the robot (i.e., its sensors, actuators, and mechanical design), the environment it is operating in, and the task it is trying to achieve. Robot programming can be even more challenging when the robot has a high degree of freedom (e.g., soft robots) and is expected to operate in unpredictable environments.

Learning from demonstration (LfD) is an alternative robot programming paradigm whereby a teacher guides the robot to acquire new skills without explicit programming [1]. It has been proven to be effective in achieving tasks that are easy to demonstrate but difficult to hand-code (e.g., backing a trailer or picking up and handling objects with robot arms). LfD has been successfully applied to help robots acquire new skills in many applications [2,3,4]. To date, almost all LfD paradigms have focused on human–robot interactions, and to the best of our knowledge, there are very few studies of robots learning from other animals (such as fish) in an automated fashion.

In recent years, navigation strategies used by animals have led to new control architectures that are resource efficient (i.e., do not require much computational power and memory usage), adaptive, and capable of generating a rich repertoire of behaviours with relatively simple algorithms [5,6,7,8,9,10,11,12,13,14,15,16]. In addition, these control architectures help to generate new hypotheses related to how animals make decisions in dynamic environments [17,18].

The work described here lays the groundwork towards developing an LfD framework to create fish-inspired robot controllers. Learning from fish poses several key challenges that remain to be addressed, which makes this research timely and exciting. A few of these challenges are how to interface between the demonstrator fish and the learner robot, recognise what to learn and when to learn, deal with complex, sub-optimal, or unsuccessful demonstrations, extrapolate the acquired information when demonstrations do not cover the entire problem space, and evaluate the performance of the robot.

The organisation of the paper is as follows. Section 2 details the proposed LfD framework, describes its core modules, and draws attention to existing design challenges. Section 3 describes the experimental protocols and data analysis methods that were used to demonstrate the utility of the proposed LfD framework. Section 4 describes the current progress in automatic fish tracking and analysis of fish trajectories. It also presents two data-driven robot controllers obtained from fish movements and evaluates their performance against two hand-coded benchmark controllers. Section 5 summarises the achievements of the presented study, highlights its limitations, and lays out a research plan for future work.

## 2. Learning from Demonstration Framework

The proposed robot training framework aims to transfer knowledge from fish to robots without explicit programming. We assume that although fish and robots differ in their sensing and movement abilities (e.g., fish have two eyes with a wide angle view versus robots who have a single front facing camera with a normal lens or fish manoeuvring and accelerating quicker than robots), there is enough overlap between the two systems so that robots can acquire the desired behaviour successfully.

The framework consists of six core modules, (1) task demonstration, (2) fish tracking, (3) analysis of fish trajectories, (4) acquisition of robot training data, (5) generating a perception–action controller, and (6) performance evaluation, which are detailed below.

### 2.1. Module 1—Task Demonstration

Two-dimensional movements of a fish are recorded using a single overhead camera. We assume that during task demonstration, the fish does not roll, pitch, or move up and down in the water column (i.e., movements are restricted to the horizontal plane perpendicular to the axis of the camera’s field of view). We also assume that the camera has enough spatial and temporal resolution to capture the desired behaviour (e.g., high frame rate for fast movements).

### 2.2. Module 2—Fish Tracking

The video recordings are digitised to estimate the position and heading angle of the fish in successive frames. Given that manual tracking (i.e., a researcher going through each video frame by frame to annotate the fish) is laborious and time consuming, automatic tracking is desired for a fast and effective means of long-term data collection.

However, this is not a trivial task due to the experimental setup. The fish appears relatively small in the captured videos. Its body shape changes dynamically during body–caudal fin undulations. It is partially (or not) visible while passing through a doorway and hiding under the plant or water filter. Light reflecting off the water distorts how it looks. Ripples generated by the air filter cause motion artefacts. Last but not least, the colour and shape of the rocks and pebbles in the background look similar to the fish.

The preliminary investigation with standard computer vision techniques (e.g., appearance-based and motion-based trackers) failed to achieve a high tracking performance. Hence, one of the main research objectives of this study was to investigate whether training an artificial neural network could lead to better tracking performance. The desired output was an assumption-free automatic tracker that could generalise well to fish with different sizes, shapes, and colours with minimal parameter tuning.

### 2.3. Module 3—Analysis of Fish Trajectories

The fish’s trajectories are first pre-processed to reduce digitisation noise, remove outliers, and handle missing data. They can then be analysed to identify successful trials, recognise multiple behaviours, associate the fish’s movements to the key features found in the environment, and track how they change over time in the short term (within a trial) and long term (across trials).

### 2.4. Module 4—Acquisition of Robot Training Data

The link between the fish and the robot is established through path following. The robot initially follows the trajectories of the fish blindly. During this process, it makes connections between its own sensory readings and actions taken. It then uses these connections to make autonomous decisions. In the past, a similar approach was used to establish a link between a human demonstrator and a mobile robot to achieve low level behaviours such as wall and corridor following and door traversal [19].

The extracted fish trajectories are translated into a set of way-points (x and y coordinates) in the robot’s environment, which is the scaled model of the fish environment, while the robot is following a given fish trajectory blindly by moving along the way-points, a data logger records its sensor readings (inputs) and the desired motor commands (i.e., outputs). The acquired input–output data are used for the training and validation of perception–action controllers described in the next module.

Several methods have been proposed to drive the robot along the predetermined way-points with high fidelity (e.g., [20,21]). The one employed in this framework starts with creating a smooth path between the way-points. It then employs a closed loop controller which continuously updates the linear and angular velocity of the robot based on the difference between its desired and actual position. The difference between the desired and actual robot position is estimated in real time using an overhead camera and an automatic robot tracking algorithm.

Sometimes, the target way-points are not accessible as the maneuverability of the robot may not be as good as the maneuverability of the fish (e.g., when the way-point is too close to a wall). In these situations, a planner, which combines obstacle avoidance and path following behaviours (similar to the one proposed in [22]), can be employed to guide the robot to the next way-point safely.

### 2.5. Module 5—Generating A Perception–Action Robot Controller

After the collection of training data, a perception–action controller is trained and validated using robot learning. The level of abstraction at which the perception–action controller is realised may vary. Two examples are a low level controller which directly links raw sensor readings (e.g., camera images) to motor commands (e.g., linear and angular velocity), and a high level controller which models the robot behaviour as state transitions (e.g., turn left after reaching the landmark). In the case of the latter, it is assumed that the robot has a cognitive map of the environment (or at least has the ability to detect key environmental features and objects) and knows how to generate the necessary motor commands to achieve the desired action.

For linking the robot’s perception to action, three modelling architectures are proposed: fuzzy control systems [23,24], Armax/Narmax (linear/non-linear auto-regresssive moving average models with exogenous inputs) system identification methodology [4,25], and artificial neural networks (ANNs) [26], all of which have been successfully applied to generate effective mobile robot controllers in the past.

The fuzzy control systems, which use linguistic variables and approximate reasoning, perform well with uncertainty and noisy sensor measurements in a way consistent with the human thinking process. The Armax/Narmax methodology generates polynomial models which reveal further information about the way in which a task is achieved, the relevance of individual sensors [27], and possible ways of improving performance [28]. In this approach, the structure of the polynomial (terms included in the model) and term coefficients are estimated automatically using the steps presented in [29,30,31]. The ANNs are made from artificial neurons loosely mimicking the integration and activation properties of real neurons, and they are very good at modelling complex input–output relationships; however, they are not as transparent and interpretable as fuzzy and Narmax models. As discussed in [32], the ANNs offer a powerful tool to drive theoretical and experimental progress in systems neuroscience, and in this framework it may help identify a set of visual cues that are important to the fish.

### 2.6. Module 6—Performance Evaluation

The perception–action controllers are employed to drive the robot in the training and novel test environments multiple times, and the performance of the robot is evaluated using various metrics including accuracy, speed, shortest path, scalability, generalisability, and mimicking the fish trajectories. In addition to having a good performance, the new controller is desired to be parsimonious and tractable to advance human understanding. These features are key for theoretical analyses of robot behaviour and forming hypotheses of fish behaviour.

## 3. Materials and Methods

The utility of the framework was demonstrated using a case study. The desired behaviour was a cue-based spatial navigation which required choosing between two locations depending on the position of a visual landmark. The desired output of the framework was a perception–action controller enabling the robot to navigate to the correct location autonomously. The robot was assumed to be a differential wheeled mobile robot with two degrees of freedom controlled by linear and angular velocity commands, and have an onboard front-facing camera.

### 3.1. Fish Learning Experiments (Task Demonstration)

Three-spined sticklebacks (*Gasterosteus aculeatus*), purchased from a commercial provider (DC Freshwater Fish), were trained in a custom-built fish tank in the Marine Biology Research Laboratory at the Department of Life Sciences, Aberystwyth University. All experimental protocols were approved by the university’s Animal Welfare and Ethical Review Body and were inline with the Animal Scientific Procedures Act.

**Figure 1:**
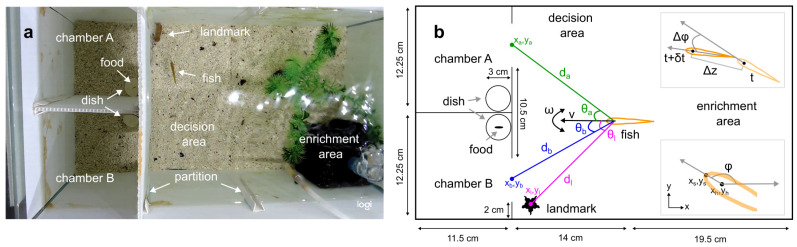
Experimental tank. (**a**). A snapshot from a learning trial as the demonstrator fish moves towards the correct chamber (i.e., chamber A) signposted by the landmark (i.e., stone). (**b**). Schematic diagram of the tank. The goal of this study was to describe the linear (ν) and angular (ω) velocity of the fish (orange) as a function of its geometric position relative to the landmark ($d_{l}$,$\theta_{l}$, pink), chamber A ($d_{a}$,$\theta_{a}$, green), and chamber B ($d_{b}$,$\theta_{b}$, blue). The inset (**bottom right**) illustrates the fish heading angle (ϕ) in the tank frame of reference, which was derived from the two digitised points along the head ($x_{s},y_{s}$ and $x_{h},y_{h}$). The inset (**top right**) illustrates the displacement (Δz) and change in heading angle (Δϕ) in δt time.

The fish tank was partitioned into four areas. On one side, there were two adjacent food chambers separated by a wall partition (Figure 1a). On the other side, there was an enrichment area where the fish would be resting in between trials, and in the middle, an open decision area connected the enrichment area to the food chambers. Food chambers had an open doorway allowing entry from the decision area, and each chamber had a small circular dish where food was presented. The dish was placed in a hidden corner and was not visible from the decision area. There was a movable wall partition separating the enrichment and decision areas. To keep fish comfortable, the entire bottom of the tank was covered by gravel substrate, and the enrichment area had an air filter and a decorative plant.

In any given learning trial, a bloodworm (food) was placed on the circular dish only in one of the chambers (i.e., the correct chamber). To reduce the risk of the fish tracking the bloodworm using olfactory cues, 5 mL liquid containing bloodworm scent was introduced to the other chamber. The landmark was placed in the corner outside the correct chamber. The trial started by removing the wall partition between the enrichment and decision areas, and a 10 min video was captured using a Logitech Webcam at 30 frames per second and at 720p resolution (ventral view).

In total, 25 fish were trained with a body length of *BL* = 4.2 ± 0.5 cm (mean ± standard deviation). Each fish went through four randomised trials per day (the correct chamber was alternated randomly between the trials) and up to 44 trials in total. In trials where fish made the wrong choice (i.e., entered the wrong chamber in their fist attempt), they were still allowed to explore the other chamber and eat the bloodworm. The fish who made the right choice in 8 out of 10 consecutive trials in less than 45 trials were regarded as the *learned* fish. Once a fish reached the learning criterion, the training was stopped.

Additional test trials were performed to confirm the targeted association between the landmark (i.e., visual cue) and the correct chamber. In these post-learning trials, the *learned* fish were tested with an unfamiliar object, and their ability to find the correct chamber was expected to drop (due to the absence of the landmark) unless they were using other sensory cues (e.g., olfactory and auditory cues) that were not accounted for. Training to criterion such as the one used in this study is widely used in animal learning experiments (e.g., [33]).

### 3.2. Fish Tracking

#### 3.2.1. Manual Video Annotations

A custom-built python script was used to annotate fish videos manually. A researcher clicked two points of interest along the head: the most anterior point (snout ($x_{s},y_{s}$)) and the midpoint between the eyes (head ($x_{h},y_{h}$)) (Figure 1b). Fish annotations were used for two tasks: (task-1) training and testing the automatic fish tracker and (task-2) deriving the perception–action robot controllers. For task-1, all 38 videos from fish-1, and 96 additional videos from the other 24 fish (the first and the last two learning trials) were annotated. On average, a 3 min of recording was digitised at five frames per second resulting in 1500 frames per video. For task-2, 27 successful trials from 14 *learned* fish were digitised (at 30 frames per second) until they entered the correct chamber. In 16 of these trials, the correct chamber was chamber B, and in 11 of them, it was chamber A. In addition, from each trial, the coordinates of the landmark ($x_{l},y_{l}$) and the entrances of chambers A ($x_{a},y_{a}$) and B ($x_{b},y_{b}$) were noted to study the fish movements in relation to these environmental features (Figure 1b).

#### 3.2.2. Training DeepLabCut Models

DeepLabCut (DLC), a marker-less pose estimation framework employing deep artificial neural networks and transfer learning, was used to train the fish tracker. In recent years, DLC (and similar) frameworks has been applied to track animals successfully in various experimental settings (indoor, outdoor, and single versus multiple animals) [34,35,36,37].

The network architecture was chosen as the ResNet-50 convolutional neural network. The input to the network was a video frame and the outputs were the predicted coordinates for both snout (${\tilde{x}}_{s},{\tilde{y}}_{s}$) and head (${\tilde{x}}_{h},{\tilde{y}}_{h}$). Two DLC models were trained and tested. The first model (hereinafter DLC-1) was trained using data from a single fish (300 frames were selected randomly from fish-1, trial-1). The second model (hereinafter DLC-2) was trained using data from all fish (50 frames were randomly selected from the first two trials of every fish). In total, 1250 frames were used to train DLC-2. Both models were trained on Google Colab over 200,000 iterations using default parameters.

#### 3.2.3. Performance Evaluation of the DLC Models

Two test datasets (dataset 1 and dataset 2) were used to evaluate the performance of the DLC models. The first test dataset included 45,065 frames from the rest of the fish-1 trials (i.e., trial-2 to trial-38, on average 1250 frames per trial). The second test dataset included 57,611 frames from the last two trials of all fish (on average 1140 frames per trial per fish).

The performance of the models was evaluated by calculating the percentage of frames (*p*), where the pose estimation errors for two points of interest, snout ($e_{s}$) and head ($e_{h}$), were both within 0.1 *BL*. This threshold was applied to remove outlier predictions where DLC models misidentified other objects in the tank as fish (e.g., wall segments, landmarks, and pebbles). The pose estimation errors ($e_{s,h}$) were calculated using the Euclidean distance,

(1) $$e_{s,h}=\sqrt{{\left(x_{s,h}-{\tilde{x}}_{s,h}\right)}^{2}+{\left(y_{s,h}-{\tilde{y}}_{s,h}\right)}^{2}}$$

The higher the *p* (best case scenario: 100%) or the lower the *e* (best case scenario: 0 *BL*), the better the performance of the tracker.

The student *t*-test was performed to compare the performance of DLC-1 and DLC-2. In addition, two researchers were asked to digitise one video trial, and the agreement between them was measured using the same error metrics. These measures indicated the inter-coder reliability, which was then used as a baseline against which the performances of the DLC models were compared. The DLC models were assumed to reach human-level performance if $p_{s,h}$ and $e_{s,h}$ were comparable to the inter-coder reliability.

### 3.3. Analysis of Fish Trajectories

#### 3.3.1. Preprocessing

Fish coordinates ($x_{s,h},y_{s,h}$) were smoothed using the Savitzky–Golay filter from Python’s SciPy library (window size = 10, polynomial order = 1) to reduce digitisation noise.

**Figure 2:**
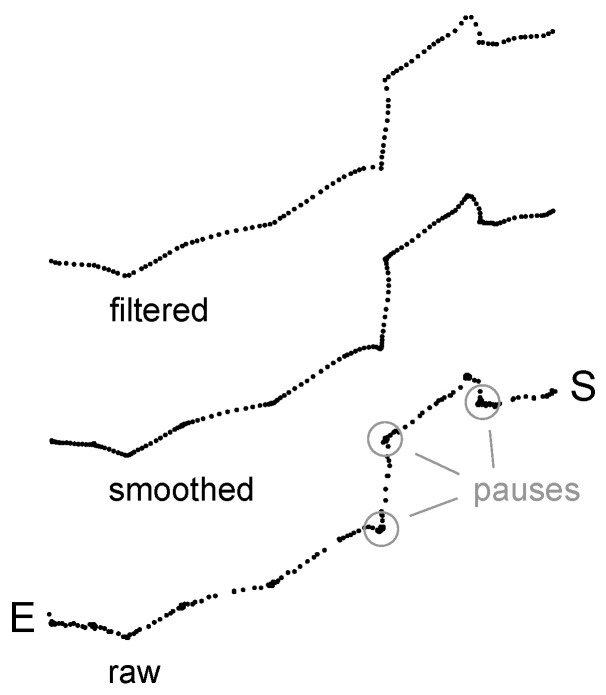
A fish trajectory from a successful trial: raw (**bottom**), smoothed (**middle**), and filtered (**top**). E and S indicate the starting and end points of the trajectory, respectively. The grey circles point to a few examples of the fish pausing.

The preliminary results from our initial investigation showed that fish movements were not continuous but rather intermittent with long pauses (Figure 2). Although these pauses might contain useful information about fish decision making (for example, see [38]), in this study, they were filtered out using a threshold for linear velocity (≤0.05 *BL* s${}^{-1}$).

#### 3.3.2. Extracting Sensor Readings and Motor Commands for the Perception–Action Controller

The acquisition of the robot training data through path following has yet to be implemented. Instead, it was reproduced from the fish data artificially by extracting the instantaneous velocity of the fish (motor commands) and their geometric position relative to the landmark and the two chambers (sensor readings) (Figure 1b).

The linear (ν) and angular (ω) velocities were calculated,

(2) $$\nu \left[n\right]=\frac{\Delta z[n]}{\delta t}$$

(3) $$\omega \left[n\right]=\frac{\Delta \phi [n]}{\delta t}$$

where Δz is the displacement between the current frame (*n*) and the previous frame (n−1) in δt time,

(4) $$\Delta z\left[n\right]=\sqrt{{\left(x_{h}\left[n\right]-x_{h}\left[n-1\right]\right)}^{2}+{\left(y_{h}\left[n\right]-y_{h}\left[n-1\right]\right)}^{2}}$$

and Δϕ is the change in heading angle, which was calculated by,

(5) $$\phi \left[n\right]=atan2\left(\left(y_{s}\left[n\right]-y_{h}\left[n\right]\right),\left(x_{s}\left[n\right]-x_{h}\left[n\right]\right)\right)$$

The fish’s geometric positions relative to the landmark ($d_{l}$,$\theta_{l}$), chamber A ($d_{a}$,$\theta_{a}$), and chamber B ($d_{b}$,$\theta_{b}$) were calculated in polar coordinates,

(6) $$d_{l,a,b}\left[n\right]=\sqrt{{\left(x_{l,a,b}\left[n\right]-x_{h}\left[n\right]\right)}^{2}+{\left(y_{l,a,b}\left[n\right]-y_{h}\left[n\right]\right)}^{2}}$$

(7) $$\theta_{l,a,b}\left[n\right]=\phi \left[n\right]-atan2\left(\left(y_{l,a,b}\left[n\right]-y_{h}\left[n\right]\right),\left(x_{l,a,b}\left[n\right]-x_{h}\left[n\right]\right)\right)$$

The sensor readings ($d_{l,a,b}$ and $\theta_{l,a,b}$) and motor commands (ν and ω) were then smoothed using the Savitzky–Golay filter (window size = 30 frames, polynomial order = 1).

### 3.4. Perception–Action Controllers

The linear velocity of the robot ($\hat{v}$) was kept constant at the average fish speed, and the focus was on controlling the angular velocity ($\hat{\omega }$),

(8) $$\begin{array}{c} \hat{\omega }=C\left({\hat{d}}_{l},{\hat{\theta }}_{l},{\hat{d}}_{a},{\hat{\theta }}_{a},{\hat{d}}_{b},{\hat{\theta }}_{b}\right) \end{array}$$

where *C* is the perception–action controller and ${\hat{d}}_{l,a,b}$ and ${\hat{\theta }}_{l,a,b}$ are the robot’s positions in polar coordinates relative to the landmark and food chambers which were computed in a similar fashion as described for fish.

Four perception–action controllers were implemented: (1) the random walk controller (a standard controller often used for exploration tasks [39]), (2) the proportional controller (a standard controller often used for goal-directed navigation tasks [40]), (3) the ANN controller, and (4) the fuzzy controller. The performance of the random walk and proportional controllers provided a benchmark while evaluating the performance of the ANN and fuzzy controllers, which were obtained empirically through the LfD framework. The weights of the ANN controller were estimated automatically from the fish data using the supervised learning paradigm. The rules and membership functions of the fuzzy controller were hand-coded after studying the fish data. Hence, the fuzzy controller can be considered as a human-filtered, fish-inspired controller.

#### 3.4.1. The Random Walk Controller

The $\hat{\hat{\omega }}$ was randomly chosen from a normal distribution,

(9) $$\hat{\hat{\omega }}=N\left(0,1\right)$$

with zero mean and one standard deviation, and was capped at ±π s${}^{-1}$. This generated a correlated random walk where the robot’s heading angle in the current frame was correlated with its heading angle in the previous frame.

#### 3.4.2. The Proportional Controller

The $\hat{\omega }$ is proportional to the heading angle relative to the correct chamber (i.e., ${\hat{\theta }}_{c}$),

(10) $$\hat{\omega }=K{\hat{\theta }}_{c}$$

The correct chamber (either A or B) was decided at the start from the robot’s heading angle; the difference in heading angle between the landmark and the correct chamber was expected to be smaller than the difference in heading angle between the landmark and the other chamber.

(11) $${\hat{\theta }}_{c}=\left\{\begin{array}{cc} {\hat{\theta }}_{a} & \left|{\hat{\theta }}_{l}-{\hat{\theta }}_{a}\right|<\left|{\hat{\theta }}_{l}-{\hat{\theta }}_{b}\right|\\ {\hat{\theta }}_{b} & \left|{\hat{\theta }}_{l}-{\hat{\theta }}_{a}\right|>\left|{\hat{\theta }}_{l}-{\hat{\theta }}_{b}\right| \end{array}\right.$$

The coefficient *K* was set to 0.12, which was determined empirically from the fish data by fitting a linear regression between ω and $\theta_{c}$ ($R^{2}$ = 0.13 and *p* < 0.01).

#### 3.4.3. The ANN controller

A multi-layer perceptron regressor network was implemented using Python’s scikit-learn and tensorflow libraries. The controller used four input variables ($d_{l}$, $\theta_{l}$, $\theta_{a}$, and $\theta_{b}$) to determine $\hat{\omega }$. The input data was standardised by subtracting the mean and dividing by the standard deviation. The controller had one hidden layer with six neurons (with the *ReLu* activation function), and the output layer had one neuron (with the *linear* activation function). During training, the fish data were doubled by multiplying $\theta_{l,a,b}$ and ω by −1 to create a more balanced dataset (to remove the bias of entering chamber B). The controller’s weights were initialised randomly by drawing from a truncated normal distribution, tuned using the stochastic gradient descent algorithm, and optimised using the *adam* solver. During training, the squared error between the desired and predicted ω was used to evaluate the performance of the network. The learning rate was set to the default value of 0.001. The batch size and number of epochs were set to 50 and 500, respectively.

#### 3.4.4. The Fuzzy Controller

**Figure 3:**
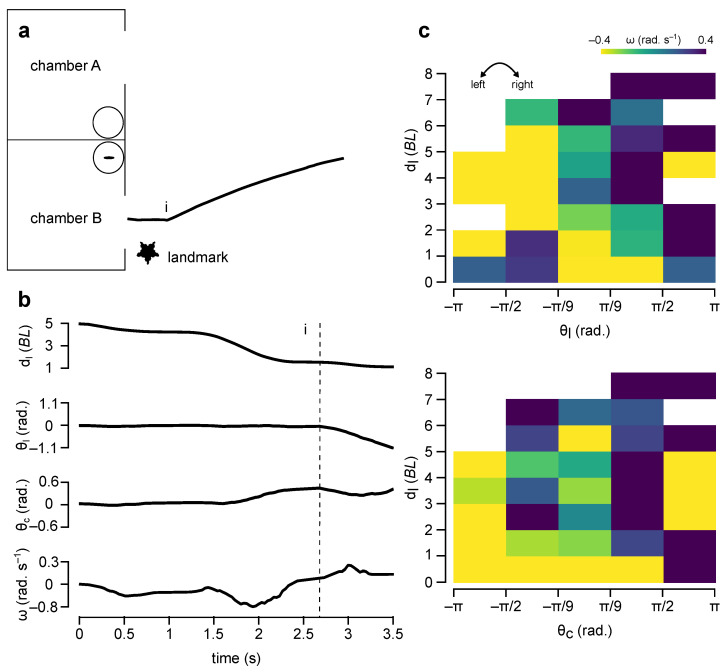
Fish data. (**a**). A sample fish trajectory (fish-2, trial-15). In this example, the fish approached the landmark first and then turned towards the chamber. (**b**). The fish’s perception ($d_{l}$, $\theta_{l}$, and $\theta_{c}$) and actions (ω) over time while moving along the trajectory are shown in (**a**). The vertical line (dashed) indicates the time point when the fish started turning towards the chamber (i.e., transition from step-1 to step-2). (**c**). Average ω (heat map) in $d_{l}$-$\theta_{l}$ and $d_{l}$-$\theta_{c}$ spaces. Warm and cool colours indicate left and right turns, respectively.

The analysis of the fish data offered a valuable insight into how to link the robot’s $\hat{\omega }$ to $\hat{d_{l}}$, $\hat{\theta_{l}}$, and $\hat{\theta_{c}}$. The preliminary results from our initial investigation suggested that some fish used a two-step algorithm to find the correct chamber: (step-1) approach the landmark and (step-2) enter the chamber nearby (for example, see Figure 3a,b). To test this hypothesis, the fish’s input space ($d_{l}$-$\theta_{l}$ and $d_{l}$-$\theta_{c}$) was partitioned into small areas and the average ω was calculated for each area.

$d_{l}$ was divided into eight equal intervals from 0 to 8 *BL* with 1 *BL* increments: (i) [0, 1], (ii) [1, 2], …, (vii) [6, 7], and (viii) [7, 8]. The numbers in square brackets indicate the lower (≥) and upper (<) boundaries of each interval. With many intervals, we hoped to identify the precise range of $d_{l}$ where the transition from step-1 to step-2 occurred.

$\theta_{l}$ and $\theta_{c}$ were each divided into five intervals between +π and −π based on the assumption that fish had a 360 degree field of view: (i) far left [−π,$-\frac{\pi }{2}$], (ii) left [$-\frac{\pi }{2}$,$-\frac{\pi }{9}$], (iii) front [$-\frac{\pi }{9}$,$\frac{\pi }{9}$], (iv) right [$\frac{\pi }{9}$,$\frac{\pi }{2}$], and (v) far right [$\frac{\pi }{2}$,π]. For this part, we had to settle for fewer number of intervals to ensure that each $d_{l}$-$\theta_{l}$ and $d_{l}$-$\theta_{c}$ area had enough data points. Although the decision for the lower and upper boundary of each $\theta_{l,c}$ interval was arbitrary, the intent was to study fish turning movements broadly when the feature of interest (the landmark or the correct chamber) was behind the fish (far left or far right), ahead but not in front of the fish (left or right), or in front of the fish (front).

The analysis of ω in these areas corroborated the two-step decision making hypothesis. When $d_{l}>2$ *BL*, fish tended to turn left when the landmark appeared on the left and vice versa, irrespective of the position of the correct chamber. When $d_{l}<2$ *BL*, fish tended to turn towards the chamber and away from the landmark (Figure 3c).

**Figure 4:**
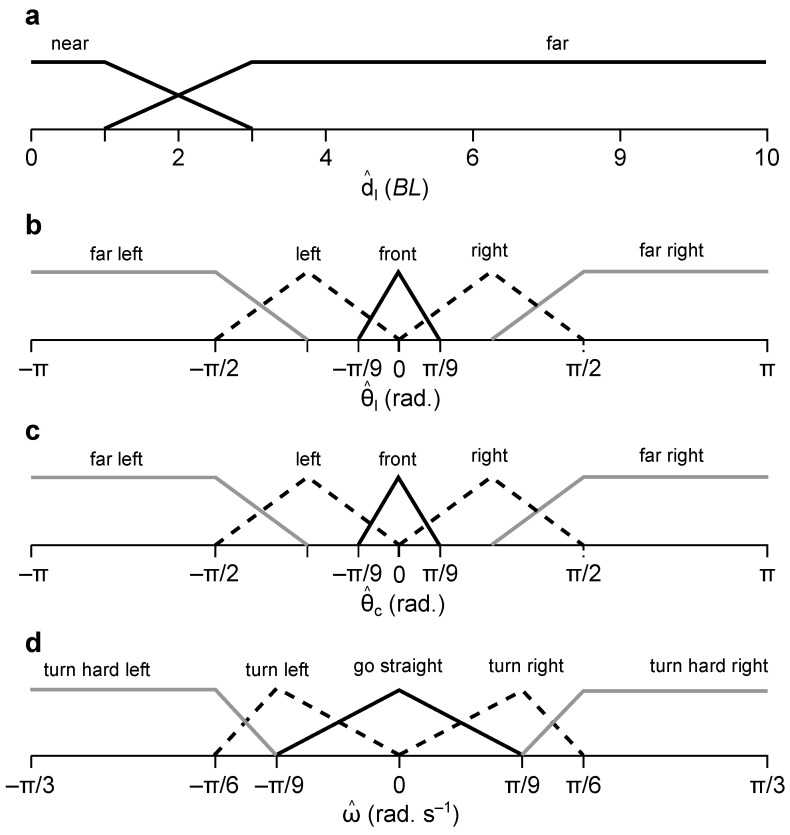
Membership functions for the input and output variables of the fuzzy controller. (**a**) ${\hat{d}}_{l}$, (**b**) ${\hat{\theta }}_{l}$, (**c**) ${\hat{\theta }}_{c}$, and (**d**) $\hat{\omega }$.

Based on these findings, we designed a type-1 Mamdani fuzzy inference system with linear membership functions and implemented it in Python using the *skfuzzy* library. The fuzzy membership functions and rules are presented in Figure 4 and Table 1, respectively. The fuzzy rules had *and* connectives, and the centre of gravity was chosen as the defuzzification method to translate rule firings to $\hat{\omega }$.

$\hat{d_{l}}$ had two membership classes (i.e., near and far) to implement the fish-inspired two-step algorithm described above. The maximum ${\hat{d}}_{l}$ was fixed at 10 *BL* based on the maximum distance that the robot could be away from the landmark. When ${\hat{d}}_{l}$ was far, $\hat{\omega }$ depended on ${\hat{\theta }}_{l}$. When ${\hat{d}}_{l}$ was near, it depended on ${\hat{\theta }}_{c}$, which was determined from the robot’s proximity to each chamber,

(12) $${\hat{\theta }}_{c}=\left\{\begin{array}{cc} {\hat{\theta }}_{a} & {\hat{d}}_{a}<{\hat{d}}_{b}\\ {\hat{\theta }}_{b} & {\hat{d}}_{a}>{\hat{d}}_{b} \end{array}\right.$$

Both ${\hat{\theta }}_{l}$ and ${\hat{\theta }}_{c}$ had five membership classes (i.e., far left, left, front, right, and far right), in line with the partitioning of $\theta_{l}$ and $\theta_{c}$. ω also had five classes (turn hard left, turn left, go straight, turn right, and turn hard right), one for each $\theta_{l,c}$ interval. The lower and upper boundaries of $\hat{\omega }$ were based on the average ω in each $\theta_{l,c}$ interval.

### 3.5. Computer Simulations

The controllers were tested using a two-dimensional particle simulation which was custom built in Python. The simulation environment had the same layout and dimensions of the fish tank (in *BL*). The position and heading angle of the particle (robot) were updated every δt=0.2 s,

(13) $$\begin{array}{c} \left[\begin{array}{c} \hat{x}\left[n\right]\\ \hat{y}\left[n\right]\\ \hat{\phi }\left[n\right] \end{array}\right]=\left[\begin{array}{c} \hat{x}\left[n-1\right]\\ \hat{y}\left[n-1\right]\\ \hat{\theta }\left[n-1\right] \end{array}\right]+\left[\begin{array}{c} \hat{\nu }cos\left(\hat{\phi }\left[n-1\right]\right)\delta t\\ \hat{\nu }sin\left(\hat{\phi }\left[n-1\right]\right)\delta t\\ \hat{\omega }\left[n-1\right]\delta t \end{array}\right] \end{array}$$

which were then used to calculate sensor readings (${\hat{d}}_{l,a,b}$ and ${\hat{\theta }}_{l,a,b}$) and subsequently the angular velocity ($\hat{\omega }$). The particle had a constant linear velocity ($\hat{\nu }$ = 1.2 *BL* s${}^{-1}$) as described in the previous subsection. During simulations, the particle had perfect sensors and motors, and could the see the landmark and the chambers from anywhere in the tank.

Two simulation experiments were run. In the first experiment, 27 simulation trials were run to replicate the fish trials, i.e., the initial conditions (the particle’s starting position and heading angle as well as the position of the landmark) were set to those measured in the fish trials. In the second experiment, 1000 simulation trials were run to measure the generalisation ability of the controllers. In each trial, the particle started from a random location in the the tank with a heading angle varying between $+\frac{\pi }{2}$ and $-\frac{\pi }{2}$ (the particle was facing the chambers when ϕ = 0), and the landmark was randomly assigned to one of the two chambers.

### 3.6. Performance Evaluation of the Controllers

A simulation trial was deemed successful if the particle went through the correct chamber first within a time threshold of 30 s without going out of bounds or colliding with the tank walls. The performance of the controllers was evaluated by measuring the percentage of successful trials (i.e., success rate, *S*). In successful trials, we also measured the time to the correct chamber (*T*) and the directedness of the path (*D*). *T* was calculated as the time difference between the start and end of the trial. *D* was calculated as the ratio between the actual path (i.e., distance travelled) and shortest path (i.e., Euclidean distance between the start and end points of the trial) (*D* ≥ 1). An analysis of variance (ANOVA) and post hoc tests were performed to compare the performance of the controllers.

## 4. Results

### 4.1. Performance of the DLC Models

The inter-coder reliability was 100 ± 0% (percentage of frames that fish were detected successfully by the two researchers) and 0.02 ± 0.01 *BL* (mean pose estimation error between the two researchers in successfully detected frames), which provided a baseline to evaluate the performance of the DLC models. The performance of the DLC models is presented in Table 2.

DLC-2 was better than DLC-1 in detecting fish, i.e., on average 4% in dataset 1 and 38% in dataset 2 (both statistically significant, p<0.01). The performance of DLC-2 was significantly lower than the inter-coder fish detection accuracy (the difference was up to 15% in dataset 1 and 10% in f dataset 2, p<0.01).

The pose estimation errors of the models were between 0.03 and 0.04 *BL*, which were significantly higher than the inter-coder pose estimation error (p<0.01). The difference between the models was not statistically significant.

**Figure 5:**
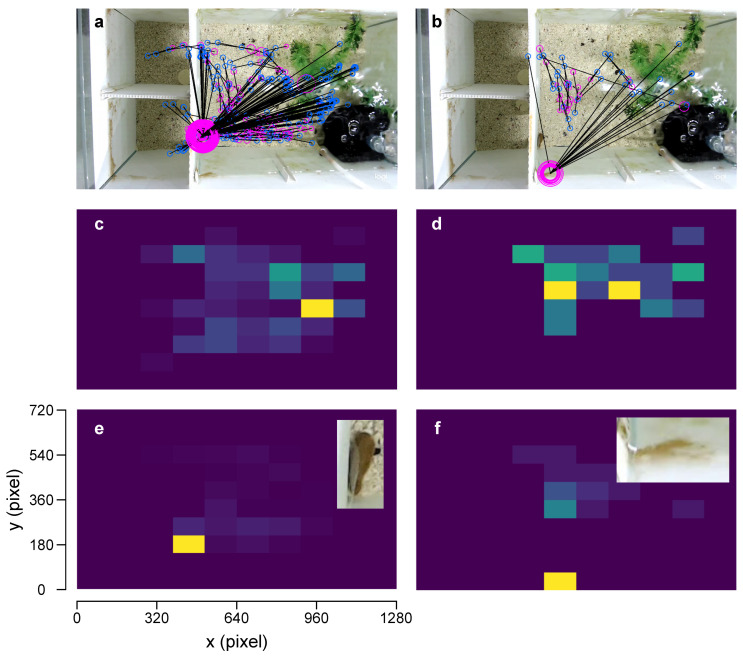
DLC model predictions with pose estimation error > 0.1 *BL* superimposed on a video image: (**a**) DLC-1 and (**b**) DLC-2 (video: fish-1, trial-37). The actual (blue) and predicted fish positions (pink). Individual data points are joined with black lines. The diameter of the predicted position markers is proportional to the pose estimation error; the higher the error, the bigger the diameter is (**c**,**d**) Heat maps showing the two-dimensional histograms of the actual fish positions when the pose estimation error of DLC-1 and DLC-2 > 0.1 *BL*. They are normalised to the number of instances (maximum value = 1), and lighter colours correspond to higher frequencies. (**e**,**f**) Heat maps showing the two-dimensional histograms of the predicted positions by DLC-1 and DLC-2 when the pose estimation error > 0.1 *BL*. Again, they are normalised to the number of instances, and lighter colours correspond to higher frequencies. Insets show the portion of the image with a high false prediction frequency.

Further investigation of the video with the lowest fish detection accuracy (fish-1 trial-37, DLC-1 = 46.6% and DLC-2 = 69.0%) revealed that the majority of large pose estimation errors (>1 *BL*), which constituted roughly 21% of DLC-1 and 3% of DLC-2 predictions, occurred when the fish was in the enrichment area (Figure 5a–d). This was likely due to occlusion (i.e., the fish was partially visible around the decorative plant) and image distortions caused by the water ripples caused by the air filter. In these instances, each DLC model converged to other areas in the tank with appearance similar to the fish (Figure 5e,f). In contrast, small errors (<1 *BL*), which constituted roughly 31% (DLC-1) and 28% (DLC-2) of incorrect predictions, mostly occurred in the decision area. In these instances, confusion between the head and tail of the fish was common.

### 4.2. Performance of the Perception–Action Controllers

Although the performance of the DLC-2 model was highly promising, the analysis of the fish trajectories and the parameter tuning of the perception–action controllers were carried out using manually digitised data points. In this way, we were able to evaluate the performance of the controllers independent of the performance of the fish tracking module. The results are presented in Table 3.

**Figure 6:**
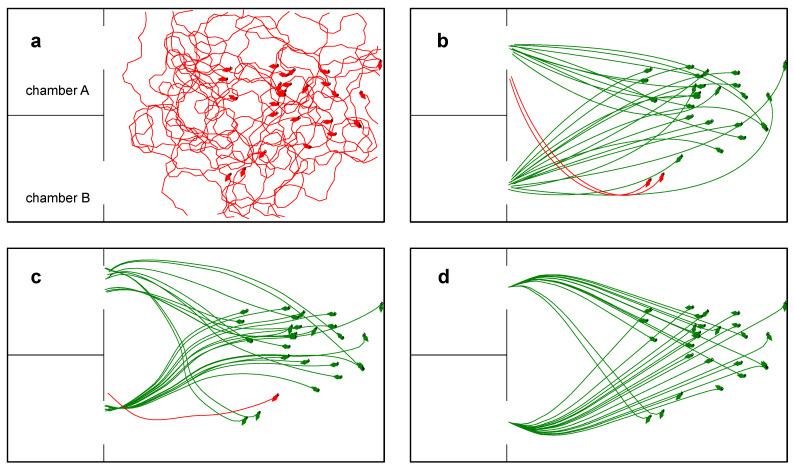
Robot trajectories from simulation experiment 1: successful trials (green) and unsuccessful trials (red). (**a**) Random walk controller. (**b**) Proportional controller. (**c**) ANN controller. (**d**) Fuzzy controller. The trajectories from the two unsuccessful trials in (**b**) suggest that the proportional controller was too slow to react when the robot’s initial heading angle was far away from the direction of the correct chamber. The trajectories from the successful and unsuccessful trials in (**c**) suggest that the ANN controller had a slight bias towards chamber A.

The proportional, ANN, and fuzzy controllers had very good performances in driving the robot to the correct chamber when the initial conditions of the simulation trials were set to the initial conditions measured in the fish trials (simulation experiment 1). The success rates for the three controllers were 93% (two unsuccessful trials), 96% (one unsuccessful trial) and 100%, respectively (Figure 6b–d). These controllers also had very similar times to the correct chamber and path directedness scores.

The fuzzy controller had a better generalisation performance than the ANN and proportional controllers when the simulation trials had random initial conditions covering a wider range of starting positions and heading angles (simulation experiment 2). The fuzzy controller’s success rate was significantly higher than the success rates of the ANN (20%) and proportional controllers (12%) (p<0.01). The fuzzy and proportional controllers had similar times to the correct chamber and path directedness scores which were significantly better the the ANN controller’s scores (p<0.01).

In both simulation experiments, the random walk controller performed very poorly with no successful trials in experiment 1 (Figure 6a) and only twelve successful trials in experiment 2.

## 5. Discussion

### 5.1. Summary

A work-in-progress LfD framework was proposed to translate fish behaviours into robot controllers automatically. The framework consists of six modules: (1) task demonstration, (2) fish tracking, (3) analysis of fish trajectories, (4) acquisition of robot training data, (5) generating perception–action robot controller, and (6) performance evaluation. Once it is complete, it is hoped that the framework will provide a research tool to study mechanisms of mapping, navigation, and learning in fish, and in return these mechanisms will help to design more autonomous and adaptive robots.

The study had two technical contributions towards improving the LfD framework. The first contribution was a DLC model (module-2) for high-throughput fish tracking. When tested on unseen video trials, the performance of the DLC model was highly promising. It detected fish successfully in 85% of the frames, and in these frames, its average pose estimation error was less than 0.04 *BL*. The human-supervised DLC tracker, where a researcher oversees the DLC predictions and makes corrections when needed, is poised to reduce the pose estimation workload at least by a factor of five.

The second contribution was the demonstration of the possibility of generating a plausible robot controller using the LfD framework. The task investigated was a cue-based spatial navigation task routinely used in learning and memory studies in fish [13]. The experimental tank had two chambers and a landmark, and the goal was to generate a controller that could autonomously drive the robot to the correct chamber signposted by the landmark.

The controllers had the robot’s current position relative to the landmark and the two chambers as the input vector (three distances and three angles) and calculated the angular velocity of the robot as the output. The controllers did not have access to the inputs and outputs from previous time steps while calculating the angular velocity. The performance of the controllers was evaluated using a two-dimensional, custom-built computer simulator. Two simulation experiments were run: one to measure the performance of the controllers in the fish trials and one to measure the generalisation ability of the controllers. In both simulations, the robot was modelled as a particle with perfect sensors and actuators and it had a constant linear velocity.

Four different controllers were created and their performance was evaluated using three metrics (success rate, time to the correct chamber, and path directedness). The first two controllers (the random walk and proportional controllers) were hand-coded and used as a benchmark to evaluate the performance of the other two controllers (the ANN and fuzzy controllers) which were generated through the LfD framework.

The random walk controller did not use any of the sensory inputs and its performance provided a likelihood estimate of entering the correct chamber by chance. Hence, it was not surprising that it did not perform well either in experiment 1 or in experiment 2. In contrast, the proportional controller did not need to rely on the position of the landmark as the knowledge of the correct chamber was revealed to the controller at the start of the trials. It used this information to drive the robot to the correct chamber in a straight-forward manner. Again, it was not surprising that the proportional controller performed well in both experiments.

The ANN controller was trained using the fish data. The network topology and the training parameters were hand-chosen but the weights were updated through an iterative process until the error between the desired and predicted angular velocity was small. The fuzzy controller was inspired from the fish data. The fuzzy rules and membership functions were hand-coded to implement a two-step algorithm: (step 1) approach the landmark and (step-2) move towards the nearest chamber.

While both the ANN and fuzzy controllers performed well in experiment 1, the performance of the ANN controller was not nearly as good as the performance of the fuzzy controller in experiment 2. Three reasons played a role in the reduced performance of the ANN controller. First, the ANN controller was not optimised. The input standardisation method, ANN topology (number of hidden layers and number of hidden neurons in each later), and training parameters (e.g., learning rate and loss function) were chosen using ad hoc methods. Second, it was likely that different fish used different navigation strategies. This was also evident in the colour maps presented in Figure 3c. There were few trials where fish moved towards the correct chamber from the start as in the proportional controller. In addition, it was likely that the fish that approached the landmark first as in the fuzzy controller had different distance thresholds while transitioning from step-1 to step-2. Third, not all fish movements were correlated with the landmark or the correct chamber. There were instances in almost every trial where fish made an opposite turn away from the landmark or the correct chamber (for instance, the first right turn in Figure 2). During training, these counter-intuitive turns might have hindered the ability of the ANN controller to converge to a more robust solution.

### 5.2. Limitations

It is not straightforward to predict fish motives. With any given fish data, it is very likely that fish turning patterns emerge from a multitude of trade-offs (e.g., staying close to the walls while exploring the space), and there would be parts not relevant to the desired task. Within the scope of automating the LfD framework (module-3 and module-4), deciding when to imitate and what to imitate without human intervention is the next step for training more robust perception–action controllers. However, we are also aware that removing parts from the fish data based on an arbitrary criterion may introduce undesired bias into the training.

The LfD framework is currently missing a controller to drive the robot along the fish trajectories (module-4). This step is critical for collecting training data with actual sensors and actuators. The new training data will require further research on how to detect the key environmental features (e.g., the landmark and the two chambers) and estimate their positions reliably. Assuming that the robot’s sensor is a front-facing camera, one plausible approach for position estimation is to track how the appearance of these feature changes in relation to the robot’s movements. For instance, the landmark would appear bigger as the robot is approaching it and would move towards the centre of the field of view as the robot is turning towards it.

The rules and membership functions of the fuzzy controller were derived manually from the fish data (module-5). Generating them automatically would also make the LfD framework less human dependant.

### 5.3. Future Work

There is work already being carried out to address the limitations mentioned above. In addition, the performance of the fish tracker will be further improved by developing a new post-processing algorithm for outlier detection and imputation. The algorithm will combine spatial (e.g., the distance between the snout and midpoint between the eyes) and temporal (e.g., the displacement of the snout between two consecutive frames) information to calculate the confidence of pose estimations. The pose estimations with a high confidence will be used to recalculate the pose estimations with low confidence based on the contextual data (e.g., fish position and velocity in previous time steps).

To date, the performance of the controllers has been evaluated using metrics focusing on accuracy and efficiency. Measuring the path similarity (i.e., how the trajectories of the robot resemble the trajectories of the fish) may provide additional insight into the controllers’ suitability for modelling fish decision making. In addition, we are planning to perform further performance evaluations: (1) after parameter tuning (e.g., we hypothesise that increasing the coefficient *K* in the proportional controller or introducing a wall collision penalty while training the ANN controller will improve their generalisation performance in experiment 2), (2) using theoretical methods to confirm the asymptotic stability of the controllers under different experimental conditions, (3) with imperfect sensors and actuators (by injecting noise in the simulator), (4) using robot simulators with more realistic sensors, actuators, and environmental conditions, and (5) by conducting experiments with real robots.

Last but not least, we have started analysing the pectoral fin and undulatory movements of the fish to better understand the mechanisms of how they change speed and direction during swimming. Analysing the whole body kinematics of fish is important for quantifying motion constraints, studying head movements and their role in locomotion and sensing [12], and designing better modular fish robots [41,42].

## Figures and Tables

**Table 1:** Fuzzy rules.

if $\hat{d_{l}}$ is far and ${\hat{\theta }}_{l}$ is far left, then $\hat{\omega }$ is turn hard left
if $\hat{d_{l}}$ is far and ${\hat{\theta }}_{l}$ is left, then $\hat{\omega }$ is turn left
if $\hat{d_{l}}$ is far and ${\hat{\theta }}_{l}$ is front, then $\hat{\omega }$ is go straight
if $\hat{d_{l}}$ is far and ${\hat{\theta }}_{l}$ is right, then $\hat{\omega }$ is turn right
if $\hat{d_{l}}$ is far and $\hat{\theta_{l}}$ is far right, then $\hat{\omega }$ is turn hard right
if $\hat{d_{l}}$ is near and ${\hat{\theta }}_{c}$ is left, then $\hat{\omega }$ is turn hard left
if $\hat{d_{l}}$ is near and ${\hat{\theta }}_{c}$ is left, then $\hat{\omega }$ is turn left
if $\hat{d_{l}}$ is near and ${\hat{\theta }}_{c}$ is front, then $\hat{\omega }$ is go straight
if $\hat{d_{l}}$ is near and ${\hat{\theta }}_{c}$ is right, then $\hat{\omega }$ is turn right
if $\hat{d_{l}}$ is near and ${\hat{\theta }}_{c}$ is far right, then $\hat{\omega }$ is turn hard right

**Table 2:** DLC results: *p* is the percentage of video frames in which fish were detected successfully and *e* is the pose estimation error in successfully detected frames for two points of interest along the head, i.e., snout (s) and in between the eyes (h). All results are reported as means ± standard deviation of the mean. The minimum and maximum values are also reported in square brackets.

	Dataset 1	Dataset 2
DLC-1		
$p_{s}$ (%)	84.9 ± 9.7 [45.4, 95.3]	48.0 ± 24.9 [1.0, 84.9]
$p_{h}$ (%)	86.2 ± 9.1 [46.7, 97.0]	47.6 ± 24.9 [0.6, 86.2]
$e_{s}$ (*BL*)	0.038 ± 0.002 [0.039, 0.042]	0.040 ± 0.005 [0.033, 0.052]
$e_{h}$ (*BL*)	0.035 ± 0.002 [0.032, 0.042]	0.035 ± 0.002 [0.030, 0.041]
DLC-2		
$p_{s}$ (%)	89.7 ± 6.8 [54.0, 97.3]	85.7 ± 5.3 [73.6, 92.7]
$p_{h}$ (%)	90.3 ± 6.9 [54.2, 98.6]	85.9 ± 5.6 [69.6, 95.5]
$e_{s}$ (*BL*)	0.032 ± 0.002 [0.029, 0.034]	0.040 ± 0.004 [0.034, 0.048]
blue$e_{h}$ (*BL*)	0.030 ± 0.002 [0.027, 0.034]	0.038 ± 0.003 [0.033, 0.044]

**Table 3:** Performance of the controllers in two simulation experiments: (1) 27 trials in total and (2) 1000 trials in total. S: success rate, T: time of travel, and D: directedness. T and D were calculated using the successful trials only. In the simulation experiment 1, it was not possible to calculate the T and D for the random walk controller as it failed in all trials. All results (except for S) are reported as means ± standard deviation of the mean.

Controller	Simulation Experiment 1			Simulation Experiment 2		
	**S (%)**	**T (s)**	**D**	**S (%)**	**T (s)**	**D**
random walk	0	nan	nan	1.2	12.4 ± 4.0	2.27 ± 0.89
proportional	93.0	6.8 ± 1.3	1.04 ± 0.04	86.4	6.9 ± 1.5	1.06 ± 0.05
ANN	96.3	6.9 ± 1.2	1.05 ± 0.01	78.0	7.4 ± 1.5	1.10 ± 0.10
fuzzy	100	6.8 ± 1.1	1.05 ± 0.02	98.7	6.8 ± 1.5	1.05 ± 0.02

## Data Availability

The data are available on request.
